# Electroacupuncture: A Feasible Sirt1 Promoter Which Modulates Metainflammation in Diet-Induced Obesity Rats

**DOI:** 10.1155/2018/5302049

**Published:** 2018-10-22

**Authors:** Dan Luo, Li Liu, Feng-xia Liang, Zhao-min Yu, Rui Chen

**Affiliations:** ^1^Department of Integrated Traditional Chinese and Western Medicine, Union Hospital, Tongji Medical College, Huazhong University of Science and Technology, Wuhan 430022, China; ^2^Department of Pathology, Wuhan No. 1 Hospital, Wuhan 430022, China; ^3^Department of Acupuncture and Moxibustion, Hubei University of Traditional Chinese Medicine, Wuhan 430061, China

## Abstract

It is generally accepted that metainflammation, a state of chronic and low-grade inflammation in obesity, plays a great role in metabolic disorder like insulin resistance. To gain further insight into the mechanism of metainflammation and find feasible therapy of obesity, diet-induced obesity (DIO) rats model and Electroacupuncture (EA) treatment were established in this trail. The results indicated that rising Lee's index, hyperlipidemia, insulin resistance, and increasing inflammation factors including NF-*κ*B, TNF-*α*, and Macrophages 1 were determined in DIO rats while EA is exhibiting an effective intervention. Furthermore, to clarify this phenomenon and provide new recognition of alternative medicine for the treatment of metainflammation, we found that EA activating Sirt1 and Sirt1-dependent deacetylation of histone (H3K9) was the key of modulation. It should be noted that, while possible, the activating of Sirt1 could lead to deacetylation of NF-*κ*B also. In this study, the deacetylation of NF-*κ*B depended on higher level of Sirt1 than H3K9, which suggested that the deacetylation via Sirt1 in metainflammation could be specific and programmed.

## 1. Introduction

Chronic and low-grade inflammation in obesity, termed metainflammation, has been attracted much attention from academia [1]. There have been studies highlighting proinflammatory cytokines, such as tumor necrosis factor *α* (TNF-*α*) released by white adipose tissue (WAT), leading to insulin resistance (IR) [2].

The efficacy and safety of Electroacupuncture (EA) on obesity and IR have been widely demonstrated [3, 4]. A part of the explanation that EA correcting IR could lie in modulating inflammation signals [5]; however, the experimental evidence was not very sufficient. Our previous work has committed that EA provides a beneficial effect on insulin resistance in obese and diabetic db/db mice via stimulation of Sirtuin 1 (Sirt1) [6]. It was also reported that EA could upregulate Sirt1-dependent PGC-1a expression in SAMP8 mice which enhances mitochondrial biogenesis and energy homeostasis [7]. Hence, we proposed that the link between EA and Sirt1 may provide a potential mechanism to EA modulating metainflammation and IR.

In mammals, Sirt1 is one of the seven homologs of silent information regulator 2 (Sir2), which contains nicotinamide adenine dinucleotide- (NAD+-) dependent protein deacetylases and ADP-ribosyltransferases and plays a critical role in DNA damage response, metabolism, and longevity [8]. A wealth of data has showed that Sirt1 regulates glucose homeostasis [9], energy homeostasis [10], insulin sensitivity [11], and inflammation [12].

Remarkably, Resveratrol, a natural Sirt1-activator [13], has been reported to have antiobesity effects [14]. Moreover, the deacetylation of histone and NF-*κ*B was testified as the main points in the Resveratrol intervention of inflammation and IR [15].

On the basis of existing literature data, we carried out studies in an effort to find out the mechanism of EA modulating obesity. Strikingly, EA likewise induced Sirt1 expression, which could cause the deacetylation of histone and NF-*κ*B and results in inhibition of inflammation signal. In order to provide sufficient evidence, Resveratrol was performed as a control also.

## 2. Methods

### 2.1. Animals

Male, eight-week-old (200±20g) SPF Wistar rats were obtained from Hubei Province CDC (Wuhan, China). They were housed at 22°C in a controlled environment and received 12h of artificial light per day (SPF laboratory animal room). The animals had free access to water in their home cages at all times. All experiments conducted on these samples were approved by the Animal Experimental Committee of Tongji Medical College, Huazhong University of Science and Technology.

### 2.2. Experimental Design.

After 1 week for acclimation upon arrival at the facility while being fed with a regular lab chow diet, the samples were divides into four groups: Normal group (n=10), Obesity group (n=10), EA group (n=10), and Resveratrol group (n=10). Normal group received normal laboratory diet while others received high-fat diet (5.4 kcal/g, 38.5% carbonized compound, 15% protein, and 46.5% fat) [16] which continued throughout the experimental process for 16 weeks. EA and Resveratrol treatment carried out from the 8th week to the 16th week, three times a week.

### 2.3. EA Delivery

During EA treatment, the rat was moderately bound by a piece of self-made clothing and hung approximately 0.15m high so that the movement of its body was restrained, but its head could move freely. EA was applied at the acupuncture points of Zusanli (ST36), Fenglong (ST40), Zhongji (CV3), and Guanyuan (CV4) using 0.30×25mm needles (Suzhou Acupuncture & Moxibustion Appliance Co, China). Needles at CV3 and CV4 were linked with two electrodes of one output while ST36 and ST40 at one side were linked with two electrodes of another output of an electrostimulator (LH202H, HANS Electronic Apparatus). The points were electrically stimulated with continuous wave of 2Hz. Intensity was adjusted to produce local muscle contractions that varied from 1mA for 10min. The acupoints selection was based on the theory of “strengthening vital Qi to eliminate pathogenic factors” in traditional Chinese medicine and our previous research [6].

### 2.4. Resveratrol Treatment

95% Resveratrol was purchased from Sigma-Aldrich (V900386, Sigma-Aldrich) and administered by gavage with the dose of 200mg/kg per time [17].

### 2.5. Lee's Index

The body weights and body lengths were measured at zero, 8, and 16 week. Then, Lee's index [body weight (g)1/3×1,000/body length (cm)] was calculated [18].

### 2.6. Fasting Blood Glucose and Postprandial Blood Sugar

Fasting blood glucose (FBG) and postprandial blood sugar (PBG) were measured at 8 and 16 week using a glucose testing machine and corresponding cartridge (Johnson & Johnson Medical Equipment Co. Ltd, Shanghai).

### 2.7. Intraperitoneal Insulin Tolerance Test and Intraperitoneal Glucose Tolerance Test

Intraperitoneal insulin tolerance tests (IPIT) were performed after six weeks of treatment. After 12h of fasting, an insulin solution of 0.5U/kg of body mass was injected intraperitoneally into the rats; blood samples were collected for glucose determination prior to insulin administration and after zero, 30, 60, 90, and 120 min. Intraperitoneal glucose tolerance tests (IPGT) were performed seven weeks following the series of treatments. Meanwhile, rats which were allowed to fast for 12h received an intraperitoneal injection of glucose (2g/kg body mass), and blood samples were collected for glucose level determination at zero, 30, 60, 90, and 120 min following glucose injection. After insulin or glucose administration, blood glucose was assayed from 10*μ*L of blood collected from the tip of the tail vein.

### 2.8. Serum FFA, Triglyceride, and Total Cholesterol

At the end of this study, blood was collected from the tip of the heart after 10% chloral hydrate for anesthesia and centrifuged at 2000-3000rpm for 10min at 4°C. Serum FFA, triglyceride (TG), and total cholesterol (TC) were analyzed by assay kits (Nanjing Jiancheng bioengineering Institute, China).

### 2.9. Real-Time Reverse Transcriptional Polymerase Chain Reaction

Rats were sacrificed at the end of the treatment and excised WAT for RNA extraction. The total RNA (1*μ*g) was reverse-transcribed into cDNA using the RevertAid First Strand cDNA Synthesis Kit (K1622, Fermentas). The mRNA expression was then quantified by real-time quantitative PCR (RT-qPCR), using the SYBR green PCR kit (DRR081A, TAKARA) and the 7900HT real-time system (7900HT Sequence Detector, ABI PRISM). Specific primers used for PCR are listed in Table 1.

Cycle threshold (Ct) data were normalized using glyceraldehyde-3-phosphate dehydrogenase (GAPDH), and the relative gene expression was calculated using the 2-^ΔΔCt^ method [19].

### 2.10. Western Blotting

WAT were homogenized in Radio Immunoprecipitation Assay (RIPA) lysis buffer and centrifuged at 12,000 rpm for 15 min at 4°C, followed by determination of protein concentration in supernatants. Protein lysates were separated by 10% SDS-PAGE gels and then electrophoretically transferred onto PVDF membranes. The membranes were blocked for 1h with 5% nonfat dry milk and then probed with primary antibodies against Sirt1 (13161-1-AP, Proteintech, China), NF-*κ*B (10745-1-AP, Proteintech, China), ac-NF-*κ*B (ab19870, Abcam, UK), TNF-*α* (60291-1-Ig, Proteintech, China), ac-H3K9 (ab10812, Abcam, UK), or H3K9 (1791, Abcam, UK) overnight at 4°C, followed by incubation with appropriate HRP-conjugated secondary antibody (Proteintech, China) for 2h at 37°C. Blots were developed using enhanced chemiluminescence and the density of the specific bands was quantified with ImageJ software (Rawak Software, Inc. Germany) and normalized to GAPDH.

### 2.11. Double Staining Immunohistochemistry

The paraffin-embedded sections from each group were randomly selected for identifying Macrophage 1 (M1) and Macrophage 2 (M2). Sections were analyzed for the specific marker of M1 phenotype (CD68/CD11C) and the marker of M2 phenotype (CD68/CD206). Briefly, after deparaffinizing, rehydrating, and washing, the sections were treated with 3% hydrogen peroxide in PBS for 10 min at room temperature to block endogenous peroxidase activity. For immunofluorescence double staining, the sections were incubated for 60 min with an antibody directed against CD68 (Abcam, UK); CD11C (Abcam, UK); CD206 (Abcam, UK). The secondary antibodies were goat anti-mouse fluorescein-conjugated antibody (Proteintech, China) or goat anti-rabbit fluorescein-conjugated antibody (Proteintech, China), applied for 1 h at room temperature. All images were obtained using a fluorescence microscope (Olympus).

### 2.12. Statistical Analysis

The values were expressed as the mean ± standard error of the mean (SEM). Statistical Package for the Social Sciences (SPSS) 20.0 software was used for the analysis of data. If data followed normal distribution and homogeneity of variance test, ANOVA was performed; if data did not follow a normal distribution and homogeneity of variance test, rank-sum test was performed for measurement of data from multiple groups. A P value of less than 0.05 was considered statistically significant.

## 3. Results

### 3.1. Effects of High-Fat Diet and EA on Lee's Index

**Figure 1:**
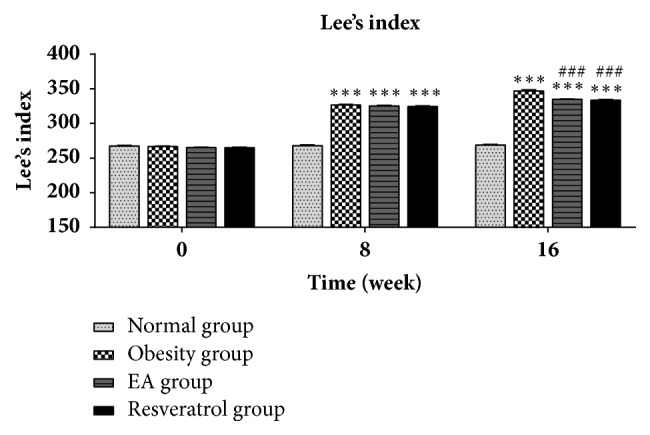
Effects of high-fat diet and EA on Lee's index. Compared with Normal group, high-fat diet significant increased Lee's index in rats form Obesity group (*∗∗∗*P<0.001 versus Normal group), EA group (*∗∗∗*P<0.001 versus Normal group), and Resveratrol group (*∗∗∗*P<0.001 versus Normal group). It was noted that Lee's index was decreased after EA (###P<0.001 versus Obesity group) or Resveratrol treatment (###P<0.001 versus Obesity group).

When compared with Normal group, Lee's index in rats form Obesity group was significant increased. Following administration of EA, Lee's index were significantly lower compared with those observed in Obesity group (Figure 1).

### 3.2. Effects of High-Fat Diet and EA on Fasting Blood Glucose and Postprandial Blood Sugar

**Figure 2:**
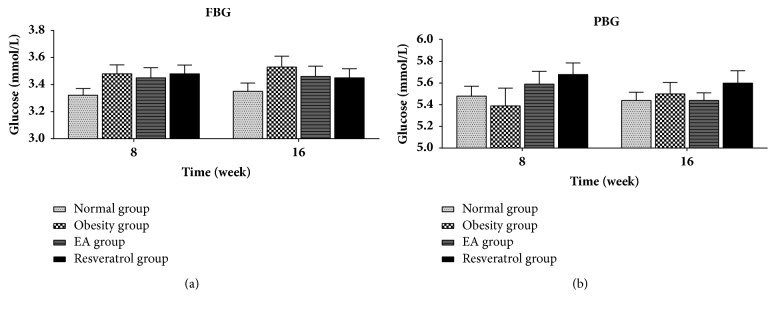
Effects of high-fat diet and EA on fasting blood glucose and postprandial blood sugar. There was no statistically significant difference between each two groups according to FBG or PBG test.

All groups had no statistical differences in FBG and PBG throughout the experimental process (Figure 2).

### 3.3. Effects of High-Fat Diet and EA on Serum FFA, Triglyceride, and Total Cholesterol

**Figure 3:**
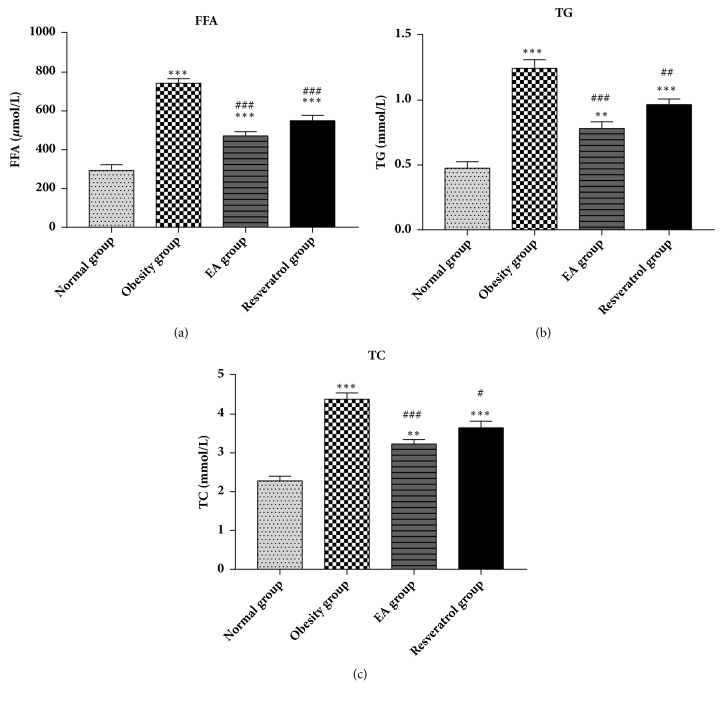
Effects of high-fat diet and EA on serum FFA (a), triglyceride (b), and total cholesterol (c). High-fat diet significant increased the serum lipid level, *∗∗∗*P<0.001 and *∗∗*P<0.01 versus Normal group. When compared with Obesity group, hyperlipemia in EA or Resveratrol group was modulated, ###P<0.001, ##P<0.01, and #P<0.05 versus Obesity group.

After modeling, there was hyperlipemia in models. It was observed that EA treatment lasting eight weeks was suitable for lowering serum FFA, TG, and TC (Figure 3).

### 3.4. Effects of High-Fat Diet and EA on IPIT and IPGT

**Figure 4:**
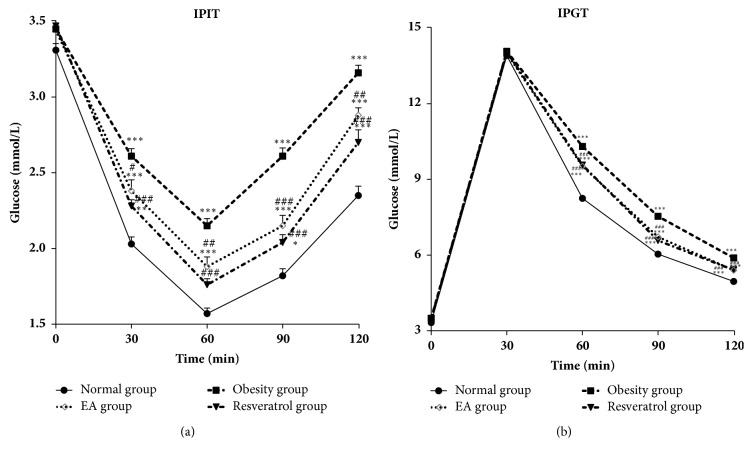
Effects of high-fat diet and EA on IPIT and IPGT. (a) In IPIT, the blood glucose concentration of rats in the Obesity, EA, and Resveratrol group at 30, 60, 90, and 120 min was higher than that observed in the normal rats, *∗∗∗*P<0.001, *∗∗*P<0.01, and *∗*P<0.05 versus Normal group. When compared with Obesity group, the insulin sensitivity was corrected in EA or Resveratrol group, ###P<0.001, ##P<0.01, and #P<0.01 versus Obesity group. (b) Similarly, glucose levels in rats from the Obesity, EA, and Resveratrol group were higher when compared with the Normal group following the IPGT test, *∗∗∗*P<0.001 versus Normal group. When compared Obesity group, the insulin sensitivity was corrected in EA or Resveratrol group, ###P<0.001 versus Obesity group.

Insulin sensitivity and glucose intolerance were greatly deteriorated in DIO rats. Compared with Obesity group, the glucose-lowering effects of insulin were corrected in the EA group based on both IPIT and IPGT (Figure 4).

### 3.5. Effects of High-Fat Diet and EA on Sirt1 Expression

**Figure 5:**
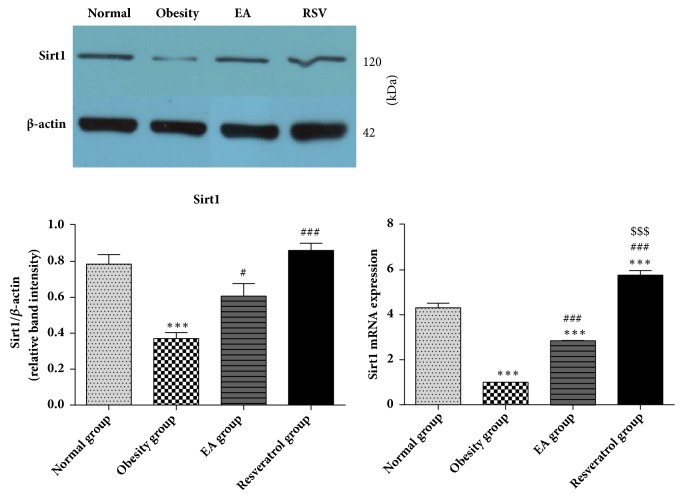
Effects of high-fat diet and EA on Sirt1 expression. In Obesity group, the protein expression of Sirt1 in WAT was lower than Normal group, *∗∗∗*P<0.001. EA or Resveratrol treatment showed a significant effect on Sirt1 activation, ###P<0.001 and #P<0.05 versus Obesity group. The mRNA level of Sirt1 was similar to the results of protein. High-fat diet suppressed Sirt1 gene expression,*∗∗∗*P<0.001 versus Normal group; EA or Resveratrol enhanced that in WAT, ###P<0.001 versus Obesity group. When compared with EA and Resveratrol group, Resveratrol demonstrated more significant effect than EA on Sirt1 activation, $$$P<0.001 and $P<0.05 versus EA group.

Downregulating the expression of Sirt1 in models was investigated and which was relieved after EA treatment. Resveratrol showed an obvious effect on Sirt1 activating than EA (Figure 5).

### 3.6. Effects of High-Fat Diet and EA on NF-*κ*B Expression

**Figure 6:**
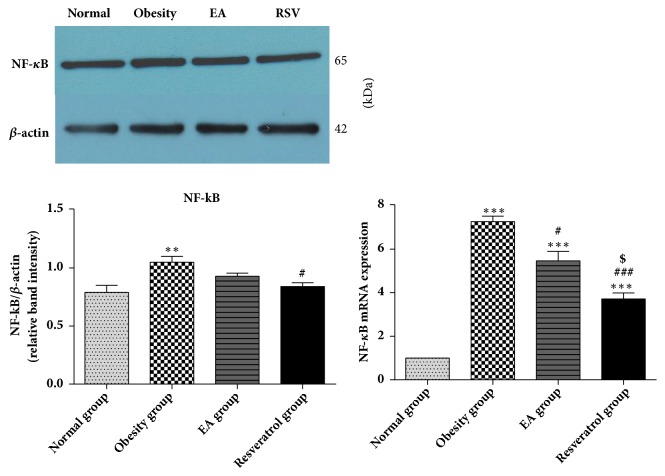
Effects of high-fat diet and EA on NF-*κ*B expression. After high-fat diet, the protein and gene expression of NF-*κ*B was enhanced in Obesity group, *∗∗∗*P<0.001 versus Normal group. Both EA and Resveratrol could downregulate the mRNA level of NF-*κ*B, ###P<0.001, and#P<0.05 versus Obesity group. Only Resveratrol group showed a significant difference in the protein level of NF-*κ*B, #P<0.05 versus Obesity group. When compared with EA and Resveratrol group, Resveratrol turned out a remarkable effect than EA in both protein and gene expression of NF-*κ*B and $P<0.05 versus EA group.

It was observed that the expression of NF-*κ*B significantly increased in DIO rats. NF-*κ*B mRNA level was significantly downregulated after EA or Resveratrol treatment, but reducing protein expression of NF-*κ*B was only founded in Resveratrol group (Figure 6).

### 3.7. Effects of High-Fat Diet and EA on TNF-*α* Expression

**Figure 7:**
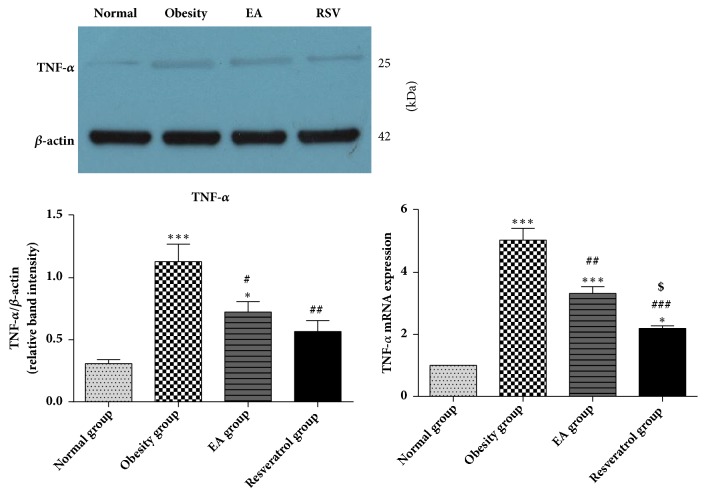
Effects of high-fat diet and EA on TNF-*α* expression. We found that the gene and protein expression of TNF-*α* significantly increased in Obesity group, *∗∗∗*P<0.001 versus Normal group. After EA or Resveratrol delivery, the increased level of TNF-*α* was modified, ###P<0.001, ##P<0.01, and #P<0.05 versus Obesity group. Resveratrol was more effective in regulation mRNA level of TNF-*α* than EA ($P<0.05 versus EA group), but there was no statistic difference in protein expression between EA and Resveratrol.

It was noticed that the gene and protein expression of TNF-*α* were significantly activated in DIO rats. EA and Resveratrol reduced both protein and mRNA levels of TNF-*α*. Resveratrol was considered as a more effective intervention than EA in downregulating mRNA level of TNF-*α* (Figure 7).

### 3.8. Effects of High-Fat Diet and EA on Sirt1-Dependent Deacetylation of Histone and NF-*κ*B

**Figure 8:**
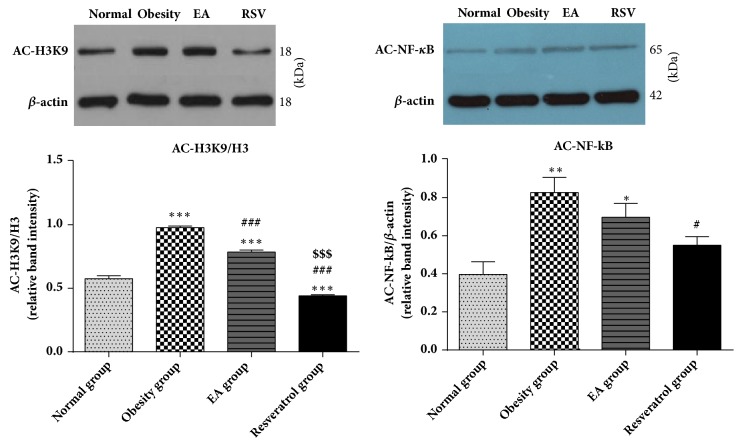
Effects of high-fat diet and EA on Sirt1-dependent deacetylation of histone and NF-*κ*B. In the results of acetylation status of H3K9, there was a great increased acetylation level in Obesity group, *∗∗∗*P<0.001 versus Normal group. It was observed that EA and Resveratrol promoted deacetylation effect, ###P<0.001 versus Obesity group. Moreover, the acetylation status of H3K9 in Resveratrol group was lower than EA ($$$P<0.001 versus EA group). When it comes to NF-*κ*B, the acetylation status was not sensitive to EA delivery. Resveratrol downregulated the acetylation status of NF-*κ*B (#P<0.05 versus Obesity group) which was enhanced by high-fat diet (*∗∗*P<0.01 versus Normal group).

To clarify the condition of Sirt1-dependent deacetylation in histone and NF-*κ*B, we measured the protein expression of acetylated H3K9 and acetylated NF-*κ*B in WAT. Increasing acetylation status of H3K9 and NF-*κ*B protein was due to the high-fat diet. After EA administration, the acetylation status of H3K9 was decreased. Although the level of AC-NF-*κ*B showed a downward trend, the difference was not statistically significant in EA group. Resveratrol enhanced both deacetylation level of H3K9 and NF-*κ*B in WAT (Figure 8).

### 3.9. Effects of High-Fat Diet and EA on Macrophages in WAT

**Figure 9:**
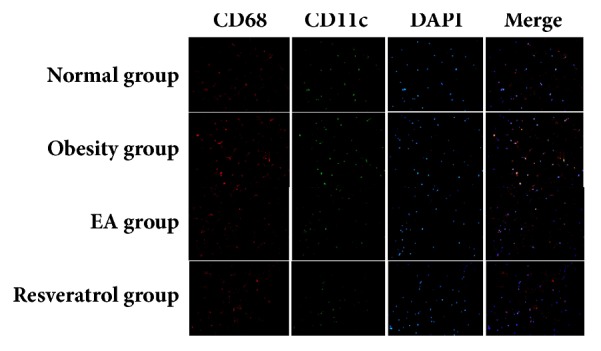
Effects of high-fat diet and EA on macrophages in WAT. Inflammatory infiltrations in rats with different treatment comprised CD68/CD11C macrophages. Immunofluorescence studies showed a decreased infiltration of CD68/CD11C M1 macrophages in the EA group and the Resveratrol group compared to Obesity group.

To determine which kind of macrophage predominantly presented in WAT. CD68/CD11C cells considered as M1 were identified by double staining immunohistochemistry. As shown in Figure 9, CD68/CD11C macrophages (M1) were dramatically decreased in in tissue from the EA or Resveratrol group, as compared to those from Obesity group. CD68/CD206 cells considered as M2 were identified by double staining immunohistochemistry. However, there was no significant difference in different group (Figure 9).

## 4. Discussion

In this study, based on DIO rats model, we evaluated the effects of 16-week high-fat diet on blood glucose, blood lipids, insulin sensitivity, glucose tolerance, and the level of inflammation factors. The research we have done suggests that high-fat diet pronounced obesity and obesity-associated metabolic problems such as insulin resistance, impaired glucose tolerance, hyperlipidemia, and metainflammation. The measured data along the DIO rats are all highly consistent with the predictions of the theoretical model. Meanwhile, Lee's index used in assessing rats' obesity was basically the same as the principle of BMI index, making it an appropriate animal model for the study of human obesity [20].

Lines of evidence have proposed EA has an application on diseases related to insulin resistance and chronic inflammation [21, 22]. In order to verify this hypothesis, EA was performed for 8 weeks. All results hinted that EA could not only correct the obesity, hyperlipidemia, and IR, but also modify the metainflammation.

To illustrate the effect of EA modulating metainflammation. Resveratrol, a widely recognized Sirt1 agonists, was used as a control. Compared with Resveratrol, EA was almost equal to the actuation of expression of Sirt1 and the curative effect of obesity was basically identical. Although Resveratrol showed more significant effect on enhancing Sirt1 expression and regulating inflammation factors, it still needs more clinic study to make sure of the efficacy and safety in human body. Combined with our previous work, all data suggested EA should be considered as a feasible Sirt1 promoter.

WAT is focused on obesity and its complications [23]. Targeting inflammation or inhibiting proinflammatory cytokines, such as TNF-*α*, is known to be metabolically beneficial [24]. Our results indicated that the activation of Sirt1 can effectively downregulate the level of TNF-*α*. The data obtained appeared to be similar to those reported earlier by other scholars [25]. Meanwhile, a plethora of recent studies have also confirmed that Sirt1 indeed inhibited the NF-*κ*B signaling [26]. In this study, we found although NF-*κ*B mRNA expression was significantly inhibited, the protein levels was not sensitive to EA treatment. Comparing EA and Resveratrol group, higher Sirt1 expression appeared to be more effective for anti-inflammation. These hypothesis agrees with a recent demonstration, in that the protein expression level of NF-*κ*B varies inversely with Sirt1 [27].

Epigenetics and obesity are increasingly valued by modern research [28, 29]. The deacetylation of histone via Sirt1 was considered an important mechanism in diseases related to inflammation [30], which could regulate the expression of NF-*κ*B [31] and TNF-*α* [32]. In this trial, we noticed that high-fat diet enhanced the acetylation status of H3K9 in WAT which was reduced after EA or Resveratrol administration. It is indicated that EA might be an effective modulation of inflammation through deacetylation of histone via Sirt1 also.

The deacetylation dependent on Sirt1 can act on many different targets, which leads to somewhat different results [33]. There were only a few researches to explain how Sirt1 could work on metainflammation. Our results demonstrated that deacetylation of NF-*κ*B was sensitive with the expression level of Sirt1 in WAT. Unlike histone, deacetylation of NF-*κ*B needs higher level of Sirt1, which indicated that deacetylation via Sirt1 to different targets could need different conditions. Despite the recent progress reviewed in microRNAs could be an important adjuster to Sirt1 [34], there was still no generally accepted theory concerning how Sirt1 could deacetylate different target.

Role of immune cells in obesity induced low-grade inflammation and IR was getting more attention nowadays. Our study found that Macrophages 1 (M1) was dramatically increased in WAT after giving high-fat diet, and it was greatly reduced after EA or Resveratrol treatment. M1 is considered as naturally triggered macrophages that produce increased levels of cytokines including IL-6 and TNF-*α*[35]. A serial of human trials has demonstrated that differentiation and migration of M1 in obesity tissues may contribute to IR [36].

The limitations of this study are clear that we have not solved all major factors in obesity like energy homeostasis problems of DIO models. Despite the great advantages about Sirt1 intervening metainflammation mentioned above there are some pitfalls. Whether there was other unknown mechanism about deacetylation via Sirt1 in WAT remains to be determined.

In conclusion, we stated that there was obvious metainflammation in DIO rats model. This modulation occurs partly through a regulation of lipid, glucose, and inflammatory responses. In particular, EA promoted the expression of Sirt1 and deacetylation of histones in WAT, introducing an effective effort to cure obesity and its complications.

## Figures and Tables

**Table 1:** 

*Sirt1*	Fw 5′-ACGCCTTATCCTCTAGTTCCTGTG-3′
	Rw 5′-CGGTCTGTCAGCATCATCTTCC-3′
*NF-κB*	Fw 5′-GAGAAGCACAGATACCACTAAGACG-3′
	Rw 5′-GTTCAGCCTCATAGAAGCCATCC-3′
*TNF-α*	Fw 5′-AGATGTGGAACTGGCAGAGGAG-3′
	Rw 5′-CACGAGCAGGAATGAGAAGAGG-3′
*Actin*	Fw 5′-CTATCGGCAATGAGCGGTTCC-3′
	Rw 5′-TGTGTTGGCATAGAGGTCTTTACG-3′

## Data Availability

The data used to support the findings of this study are available from the corresponding author upon request.
